# *Neospora caninum* infection as a cause of reproductive failure in a sheep flock

**DOI:** 10.1186/s13567-014-0088-5

**Published:** 2014-08-26

**Authors:** Marta González-Warleta, José Antonio Castro-Hermida, Javier Regidor-Cerrillo, Julio Benavides, Gema Álvarez-García, Miguel Fuertes, Luis Miguel Ortega-Mora, Mercedes Mezo

**Affiliations:** Laboratorio de Parasitología, Centro de Investigaciones Agrarias de Mabegondo, INGACAL-Xunta de Galicia, Carr. Betanzos a Mesón do Vento km 7, 15318 A Coruña, Abegondo Spain; SALUVET, Animal Health Department, Complutense University of Madrid, Ciudad Universitaria s/n, 28040 Madrid, Spain; Instituto de Ganadería de Montaña CSIC-ULE, Finca Marzanas, 24346 Grulleros, León Spain

## Abstract

*Neospora caninum* has been detected only sporadically in cases of ovine abortion, and it has therefore traditionally been considered as an unimportant parasite in small ruminants. This study was carried out with the aim of identifying the pathogen causing serious reproductive problems on a commercial sheep farm. Sera from all rams and ewes tested negative for antibodies against Border disease virus, Schmallenberg virus and *Coxiella burnetii*, and infections by these agents were therefore ruled out. Nevertheless, seropositivity to *N. caninum* and/or *Toxoplasma gondii* was detected, although the seroprevalence was higher in the case of *N. caninum*. The percentage of lambings and the number of lambs per dam were significantly lower in ewes that were seropositive to *N. caninum* while no effect on these parameters was detected in ewes that were seropositive to *T. gondii*. There was also no evidence of infection by *T. gondii* in the foetal/lamb tissues analyzed by PCR and/or immunohistopathological techniques. On the contrary, the DNA of *N. caninum* was detected in 13 out of 14 foetuses/lambs descendant from dams seropositive to this parasite. Characteristic lesions caused by *N. caninum* and/or its antigen were also detected. Genotyping of the *N. caninum* DNA revealed only two closely related microsatellite multilocus genotypes. The results clearly demonstrate that infection by *N. caninum* was the cause of the low reproductive performance of this sheep flock.

## Introduction

Reproductive failure of infectious aetiology is often identified as one of the primary causes of underperformance in ruminant livestock. The protozoan pathogens *Neospora caninum* and *Toxoplasma gondii* are often associated with this failure: *N. caninum* is currently considered the main cause of abortion and neonatal death in cattle, whereas *T. gondii* has traditionally been thought to be one of the principal agents causing abortion in sheep [1–5].

Nevertheless, research on the aetiology of ovine abortion is scarce and may not reflect the current epidemiological situation of neosporosis and toxoplasmosis, at least in some geographical areas. For many years, the aetiological diagnosis of ovine abortion induced by protozoa has been based on histopathological examination of foetal tissues. However, histopathological techniques do not enable accurate differentiation between *N. caninum* and *T. gondii*, because of the similarity in the morphology of both parasites and in the lesions induced, and therefore diagnostic errors cannot be ruled out [2,6]. Moreover, these techniques rely heavily on adequate quality and preservation of the samples, which are often deficient in abortion cases. These methodological limitations have largely been overcome by using very sensitive and specific molecular techniques, which enable detection of the parasite DNA and genetic characterization. Thus, by using PCR, Moreno et al. [7] detected similar percentages of infection by *N. caninum* and *T. gondii* in ovine aborted foetuses from different regions of Spain. Accordingly, the detection of parasite DNA in foetuses and placentas of seropositive sheep is currently considered a good method for confirming diagnosis of neosporosis and/or toxoplasmosis in sheep flocks.

Regarding the in vivo diagnosis of infected sheep, different serological techniques based on antibody detection have been used to determine the seroprevalence of both infections in different geographical areas [8–11]. Nevertheless, in most of these studies, individual reproductive parameter data were not available, and the relation between serostatus and reproductive performance could not be established. Specifically, in Galicia (NW Spain), the importance of neosporosis in sheep flocks remains largely unknown. However, it should be noted that in this region neosporosis is highly prevalent in cattle, in which postnatal infection has frequently been demonstrated [12]. As pastures and water sources are shared by sheep and cattle, an association between both host species in the epidemiology of this parasitosis is possible. Here, we provide information about the involvement of infection by *N. caninum* in reproductive failure detected during a two-year period on a commercial sheep farm. Serological, histopathological and molecular techniques were used with the aim of obtaining an accurate diagnosis. Genotyping of the *N. caninum* population implicated in the abortions occurring in the flock was also carried out.

## Materials and methods

### Farm description

The study was carried out during 2011 and 2012 on a commercial farm in which the owner had observed a dramatic reduction in the reproductive performance of sheep in the 2010 lambing season. This farm, located in the province of Lugo (Galicia, Spain), maintained a flock comprising Berrichon, Romanov and Berrichon x Romanov sheep. The animals were maintained on a grazing-based diet supplemented with conserved forage (hay and silage) and concentrate during the breeding season. Lick blocks were also provided as an additional source of minerals. The mating season lasted from January to April, and lambing was concentrated between June and September. The lambs were weaned at 4 months of age and were then sold or left in the flock as replacement animals. All sheep were annually dewormed and vaccinated against chlamydial enzootic abortion, salmonellosis caused by *Salmonella abortus ovis* and *Clostridium perfringens* enterotoxemia (Ovivac CS®, Hipra Laboratories, Barcelona, Spain; Basquin plus®, Ovejero Laboratories, León, Spain). The flock was officially certified as brucellosis free. The flock was guarded by two adult dogs. Furthermore, the farm was located in a farming area where there are many free-ranging dogs and cats, which are not always properly controlled.

### Sampling and data collection

Blood samples were collected from all ewes and rams in the flock at the beginning of the mating season in 2011 and 2012. At the same times as these samples were collected, blood and stool samples were also taken from the two guard dogs. The sera were separated by centrifugation and stored frozen until analysis for antibodies to the main pathogens that cause ovine abortion. Moreover, during this period, 4 foetuses (approximately 3 months of gestation), 15 stillborn lambs and 2 lambs born with signs of neurological disorders were submitted to the laboratory, where necropsy was performed. Samples of different tissues (brain, liver, heart, lung and placenta) were collected when available and suitable for subsequent analyses (PCR, histology and immunohistochemistry). A sample of each organ was fixed in 10% buffered formalin and the remainder of the tissue was preserved at −20 °C.

The following reproductive data were provided each year from the farm owner: 1) number of ewes that lambed and number of lambs born; 2) number of ewes with visually confirmed foetal expulsion; 3) number of ewes with no gestation product detected. The viability of the lambs was also registered, differentiating between viable lambs (alive three days after birth) and non-viable lambs (stillborn and dead within three days of birth). Moreover, information about the pedigree of each ewe was obtained in order to match the mothers to their daughters.

### Serology

The status of the flock for the main infections causing ovine abortion was determined by detecting specific antibodies in the serum from ewes and rams. The presence of specific antibodies against Border disease virus, Schmallenberg virus and *Coxiella burnetii* was determined by the respective ELISA tests (commercially available from IDEXX Laboratories, Barcelona, Spain). Anti-*T. gondii* antibodies were detected by using a latex agglutination test (Mast Toxoreagent test®; Mast Group Ltd, Bootle, UK). All tests were performed following the manufacturer’s instructions.

Anti-*N. caninum* antibodies were detected both in sheep and dogs. For the sheep, an in-house ELISA was used, with the soluble antigens prepared from tachyzoites of Nc-1 isolates as target [13], as described by Álvarez-García et al. [14]. Briefly, the assay involved the following steps: 1) coating microtitre plates with 100 μL/well of antigen solution (5 μg protein/mL); 2) addition of duplicate serum samples (100 μL) diluted 1:100 in phosphate buffer saline (PBS) with 0.2% Tween 20 (PBS-T) and 1% dry skimmed milk; 3) incubation with peroxidase conjugated anti-ovine monoclonal IgG (Sigma-Aldrich, Madrid, Spain) diluted 1:20 000 in PBS-T (100 μL/well); 4) addition of substrate (OPD, Sigma-Aldrich, Madrid, Spain), and reading the optical density (OD) at 492 nm. The plates were washed with PBS-T and incubated at 37 °C. In each plate, samples of the same positive and negative control sera were tested. The results were expressed as percentage of positivity (PP = (OD sample - OD negative control)/(OD positive control - OD negative control) × 100). The cut-off PP (7.0%) was calculated as the mean plus 3 SDs of the PPs for sheep sera (*n* = 100) collected from neosporosis-free flocks.

For the dogs, a direct agglutination test was used to detect antibodies to *N. caninum* [15]. In addition, faecal samples from the dogs were processed by a biphasic concentration technique and the resulting samples were examined by light microscopy for detection of protozoan oocysts [16].

### Detection of parasite DNA by PCR

Genomic DNA was extracted from 20–50 mg of foetal tissue samples using the commercial Maxwell® 16 Mouse Tail DNA Purification Kit, developed for automated Maxwell® 16 System (Promega, Wisconsin, USA), following the manufacturer’s recommendations. The concentration of DNA for all samples was adjusted to 50–100 ng/μL. *T. gondii* DNA was detected by a nested-ITS1 PCR adapted to a single tube, with the primers previously described by Hurtado et al. [17]. *N. caninum* DNA was detected by an adapted single-tube nested-ITS1, with the external and internal primers used by Buxton et al. [18], as previously described [19]. Each *T. gondii* and *N. caninum* PCR reaction was performed in a final volume of 25 μL under PCR conditions described by Hurtado et al. [17].

In the first step, all the brains were analyzed by PCR techniques specific for *N. caninum* and *T. gondii*, and when some of these tested positive for parasite DNA, the other available tissues (liver, heart and lung) from the corresponding foetuses/lambs were also analyzed to determine the tissue distribution of the protozoa. Two-three different DNA extractions were performed with each tissue sample and tested by PCR. Negative controls, including reactions with no template and DNA samples from non-infected foetuses, were included in each round of DNA extraction and PCR. Positive PCR controls with a quantity of *T. gondii* or *N. caninum* genomic DNA equivalent to 10 and 1 tachyzoites were also included in each batch of amplifications. PCR products were visualized under UV light in 1.5% agarose/ethidium bromide gel.

### *Neospora caninum* genotyping

DNA samples obtained from *N. caninum* PCR-positive brains were used for genotyping by multilocus microsatellite analysis. Specifically, MS4, MS5, MS6A, MS6B, MS7, MS8, MS10, MS12 and MS21 markers were amplified using specific primers and nested-PCR conditions as described by Regidor-Cerrillo et al. [20]. The sizes of the PCR products for all microsatellites were determined in a 48-capillary 3730 DNA Analyser (Applied Biosystems, Foster City, CA, USA) with GeneScan-500 (LIZ) size standards (Applied Biosystems) (at the Unidad Genómica del Parque Científico de Madrid), and the results were analyzed with GeneMapper1 v3.5 software. Microsatellites MS7 and MS10 must also be analyzed by sequencing because they contain, respectively, a single nucleotide polymorphism (SNP) and a complex sequence with three different repetitive trinucleotide motives, and therefore the nucleotide composition can vary without any change in the fragment length. Both markers were sequenced using a Big Dye Terminator v3.1 Cycle Sequencing Kit (Applied Biosystems) and a 3730 DNA Analyser (Applied Biosystems) (at the Unidad Genómica del Parque Cientifico de Madrid) for complete allele identification. Sequences were analysed using BioEdit Sequence Alignment Editor v.7.0.1 software (Copyright 1997–2004 Tom Hall, Ibis Therapeutics, Carlsbad, CA 92008, USA). Allele assignment was performed as previously described [20].

### Histology and immunohistochemistry

Buffered formalin fixed tissue samples from one aborted foetus, five stillborn and two newborn lambs were processed for conventional histological analysis. Tissue samples were dehydrated, embedded in paraffin, cut at 4 μm, and finally stained with haematoxylin and eosin (HE). Histological lesions found by microscopy were classified according to previous descriptions [21] as unrelated, consistent (*) or characteristic (**) lesions of protozoan infection.

Immunohistochemical detection of *N. caninum* and *T. gondii* antigens was carried out, according to a previously described protocol [22,23], on tissues (brain, liver, heart and lung) from 8 foetuses/lambs and on the placenta of one foetus. For detection of *N. caninum* antigen, sections were first treated with a commercial trypsin solution (Abcam, UK), for 15 min at 37 °C, to enhance labelling, and they were then incubated overnight with an in-house rabbit polyclonal antiserum against *N. caninum* tachyzoites [14], diluted 1:3000. Slides for *T. gondii* antigen detection were incubated immediately after dehydration with an in-house rabbit polyclonal antiserum against *T. gondii* tachyzoites, diluted 1:4000 [24]. A polymer-based detection system (EnVision + system Labelled Polymer-HRP anti-rabbit; Dako, Glostrup, Denmark) was applied for 30 min at room temperature, and the reaction was developed with 3, 3′-diaminobenzidine tetrahydrochloride (DAB Peroxidase Substrate Kit; Vector Laboratories). The sections were counterstained with Mayer’s haematoxylin. The technique was assessed by using appropriate controls (i.e. tissue sections containing cysts of *N. caninum*, *T. gondii* or *Sarcocystis* spp.). Weak reactivity between the anti-*N. caninum* antiserum and the cysts of *T. gondii* and *Sarcocystis* spp. was observed. Nevertheless, it was faint and clearly weaker than that produced by the *N. caninum* cysts. No cross-reactions were observed for the anti-*T. gondii* antiserum.

### Statistical analysis

Seropositivity to *N. caninum* and *T. gondii* infections in the two year study period was compared by χ^2^ test, while the inter-annual differences in antibody levels were assessed by using either a *t*-test, in the case of the PPs to *N. caninum*, or the Mann–Whitney *U* test, in the case of the antibody titres against *T. gondii*. In order to relate the serostatus of ewes to reproductive performance, two reproductive indexes were determined: 1) Percentage of lambing ewes = (number of ewes that lambed/number of ewes exposed to rams) × 100, and 2) Viable lamb index = number of viable lambs/number of ewes that lambed. The following tests were used: χ^2^ test (percentage of lambing ewes) and non-parametric Mann–Whitney *U* test (viable lamb index).

## Results

### Serological status for abortifacient agents

All ram sera tested negative for antibodies against the abortifacient agents analyzed. The ewe sera were also negative for antibodies against Border disease virus, Schmallenberg virus and *Coxiella burnetii*. Nevertheless, seropositivity to *N. caninum* and *T. gondii* was detected in the flock at both sampling times. In 2011, the percentage of seropositivity to *N. caninum*, *T. gondii* and both protozoa was 34.3% (91/265), 10.2% (27/265) and 5.7% (15/265), respectively (Table 1). At the end of this year, a total of 68 seropositive ewes (53 seroreactive to *N. caninum*, 6 seroreactive to *T. gondii* and 9 seroreactive to both parasites) were culled and replaced with female lambs drawn from offspring of seronegative dams, so that the flock seropositivity to both infections decreased in 2012. Accordingly, the decline in *N. caninum* mono-infection seroprevalence was sharp (from 34.3% to 17.5%) and statistically significant (*p* < 0.001), while the decrease in *T. gondii* mono-infection seroprevalence was moderate (from 10.2% to 8.6%). There was no statistically significant reduction in the seroprevalence of co-infection (from 5.7% to 2.4%). Inter-annual variations in antibody levels against *N. caninum* (PP values) and/or *T. gondii* (titre level) were not observed in any of the seropositive groups (Table 1).

**Table 1:** **Seropositivity for single and mixed infections with** ***N. caninum*** **and** ***T. gondii*** **in two consecutive years**

**Year**	**n**	***N. caninum***		***T. gondii***		***N. caninum*** **+** ***T. gondii***	
		**Seropositivity n (%)**	**PP** ^**1**^ **mean ± SD**	**Seropositivity n (%)**	**Titer** ^**2**^ **Range**	**Seropositivity n (%)**	**PP; Titer mean ± SD; Range**
2011	265	91 (34.3)^***^	70.5 ± 23.8	27 (10.2)	64-256	15 (5.7)	75.0 ± 20.5; 64-256
2012	245	43 (17.5)^3^	74.0 ± 32.2	21 (8.6)	64-256	6 (2.4)	44.9 ± 37.6; 64-128

^1^cut-off percentage of positivity (PP) for in-house ELISA = 7.0%; ^2^cut off titre for Mast Toxoreagent test® = 64; ^3^this number came from 38 seropositive ewes that remained after culling in 2011 + 5 ewes that seroconverted in 2012. ***Statistically significant at *p* < 0.001.

At the flock level, the relationship between the seropositivity of mothers and daughters to *N. caninum* was able to be assessed in 57 seropositive sheep whose dams had a known serostatus, and a positive association was observed in 53 of these (93%). In the case of *T. gondii*, this relationship was only able to be analyzed in 12 seropositive sheep, and the association between mother-daughter seropositivity was much lower (58%).

At an individual level, changes in serostatus were scarce. Indeed, none of the seropositive ewes become seronegative, and conversion to seropositivity was only observed for *N. caninum* infection in 5 ewes. The anti-*N. caninum* antibody levels in 4 of these 5 sheep were very low (between 8.8% and 14.6%), i.e. around the cut-off (7%) that discriminates between positivity and negativity.

The lambing percentage for the flock was very low (75.1%) in the first year of study. However, it increased (87.8%) in the second year, after several seropositive ewes were culled and replaced with seronegative ewes. To determine whether serostatus for *N. caninum* and/or *T. gondii* infections influenced reproductive performance, we compared the percentage of lambings and the mean number of viable lambs/dam recorded in seropositive and seronegative ewes (Table 2A and B). In 2011, the lambing percentage was significantly lower (*p* < 0.05) in the ewes that were seropositive to only *N. caninum* (59.3%) and seroreactive to both protozoa (60.0%) than in the ewes that were seropositive to only *T. gondii* (88.9%) and seronegative to the both parasites (84.8%) (Table 2A). The mean number of viable lambs produced by the *N. caninum* seropositive sheep was significantly lower (*p* < 0.05) than the mean number produced by seronegative sheep (1.8 compared with 2.2). In 2012, similar results were obtained for both reproductive parameters, although differences in the number of viable lambs were not statistically significant (Table 2B).

**Table 2:** **Impact of** ***N. caninum*** **and** ***T. gondii*** **ewe serostatus on the reproductive results of the farm in 2011 (A) and 2012 (B)**

**A**			
**Serostatus**	**n**	**Lambing ewes (%)** ^**1**^	**Viable lambs/ewe** ^**1**^
Positive to *N. caninum*	91	54 (59.3%)^a^	1.8 (99/54)^a^
Positive to *T. gondii*	27	24 (88.9%)^b^	2.2 (53/24)^ab^
Positive to *N. caninum* + *T. gondii*	15	9 (60.0%)^a^	1.8 (16/9)^ab^
Negative	132	112 (84.8%)^b^	2.2 (242/112)^b^
**B**			
**Serostatus**	**n**	**Lambing ewes (%)** ^**1**^	**Viable lambs/ewe**
Positive to *N. caninum*	43	31 (72.1%)^a^	1.9 (53/31)
Positive to *T. gondii*	21	20 (95.2%)^b^	2.1 (41/20)
Positive to *N. caninum* + *T. gondii*	6	4 (66.7%)^ab^	1.5 (6/4)
Negative	175	160 (91.4%)^b^	2.0 (322/160)

^1^Different letters within each column indicate statistical differences between serostatus groups, at *p* < 0.05.

No antibodies to *N. caninum* were detected in the sera from the dogs, which also tested negative by coprological analysis.

### Detection and genotyping of the parasite DNA present in tissues from aborted foetuses, stillborn and newborn lambs with neurological disorders

*Neospora caninum* DNA was not detected in any foetus/lamb from dams seronegative to this parasite (Table 3). On the contrary, parasite DNA was found in 13 of 14 (92.9%) descendants from ewes that were seropositive to *N. caninum*. Analysis of samples from other tissues (liver, heart and lung) showed that liver and heart were the organs in which the parasite DNA was next most frequently detected. *N. caninum* DNA was also detected in the single placenta that was retrieved (foetus number 4). All organs of lamb number 21, which was born with neurological disorders, tested positive to *N. caninum*-PCR. *T. gondii* DNA was not detected in the tissues from any of the foetuses/lambs, which is consistent with both the seronegativity of the dams and the absence of cases of seroconversion during the study period.

**Table 3:** **Results of PCR, histology and IHC in tissues of foetuses/lambs and relationship with maternal serostatus**

**Sample** ^**1**^	**Year**	**Maternal serostatus**		**Results obtained in tissues from foetuses/lambs** ^**4**^							
				**PCR**					**Histology** ^**5**^	**IHC** ^**6**^	
				***N. caninum***				***T. gondii***		***N. caninum***	***T. gondii***
		***N. caninum*** **PP** ^**2**^	***T. gondii*** **titre** ^**3**^	**Brain**	**Liver**	**Heart**	**Lung**	**Brain**	**Brain lesions**	**Brain**	**Brain**
1	2011	72.5	< 16	2/2	na	na	na	-	na	na	na
2	2011	86.3	32	2/2	-	1/2	-	-	na	na	na
3	2012	89.9	< 16	2/2	-	-	-	-	na	na	na
4^$^	2012	44.0	< 16	3/3	1/3	-	-	-	**	+	-
5	2011	65.1	< 16	2/2	2/2	2/2	-	-	na	na	na
6	2011	65.1	< 16	2/2	2/2	2/2	-	-	na	na	na
7^#^	2011	79.3	< 16	2/2	na	na	na	-	na	na	na
8	2011	0.0	< 16	-	nd	nd	nd	-	na	na	na
9	2011	52.0	< 16	2/2	-	-	-	-	na	na	na
10	2011	94.1	32	-	nd	nd	nd	-	na	na	na
11	2011	94.1	32	1/2	-	-	-	-	na	na	na
12	2011	82.1	< 16	3/3	-	-	-	-	-	+	-
13	2011	0.0	< 16	-	nd	nd	nd	-	*	-	-
14	2012	1.4	< 16	-	nd	nd	nd	-	Unrelated	-	-
15	2012	28.1	16	3/3	na	na	na	-	na	na	na
16^a^	2012	0.0	16	-	nd	nd	nd	-	na	na	na
17ª	2012	0.0	< 16	-	nd	nd	nd	-	na	na	na
18^b^	2012	0.0	< 16	-	nd	nd	nd	-	*	-	-
19^b^	2012	0.0	< 16	-	nd	nd	nd	-	-	-	-
20	2012	64.6	< 16	3/3	na	na	na	-	*	+	-
21	2012	98.9	< 16	3/3	2/3	3/3	1/3	-	**	+	-

^1^1-4: aborted foetus; 5–19: stillborn; 20–21: newborn with neurological disorders; $: In this sample, parasite was also detected in placenta, by PCR and IHC; ^#^All organs were analyzed for the presence of bacteria and viruses causing ovine abortions, and the results were negative; ^a,b^: lambs indicated by the same superscript letter were twins; ^2^Positivity Percentage (PP) as determined by ELISA (cut off ≥ 7.0%); ^3^Titre determined by agglutination (cut off ≥ 1:64);

^4^Fractions represent the number of positive tissue samples/total number of samples checked by PCR; (−): negative results; na: samples were not available or suitable for analysis; nd: not determined when the target organ (brain) was negative by PCR; ^5^Histological features were classified according to previous descriptions [21] as unrelated, consistent (*) or characteristic (**) lesions of protozoan infection.

Complete or almost complete microsatellite multilocus genotypes (MLGs) were obtained for most of *N. caninum* in foetus/lamb brain samples (Table 4). Only two microsatellite genotypes were identified in all infected samples, and these genotypes only differed in a single repeat unit in the MS5 locus. Mixed infections by different genotypes were not found in any foetus/lamb.

**Table 4:** **Microsatellite genetic profiles for the 13 infected-brain samples from foetuses/lambs**

**Microsatellite loci** ^**a**^	**Repetitive motif sequence**	**Number of fetus/lamb**												
		**1**	**2**	**3**	**4**	**5**	**6**	**7**	**9**	**11**	**12**	**15**	**20**	**21**
MS4	GC-*(AT)*_*X*_-ACATTT-(AT)_2_-AC	15	15	15	15	15	15	15	15	15	15	15	ND	15
MS5	CG-*(TA)*_*X*_-TGTA-GG	16	16	16	ND	15	15	16	16	ND	15	15	15	ND
MS6A	GC-*(TA)*_*X*_-AC	15	15	15	ND	15	15	15	15	15	ND	15	15	15
MS6B	CC-*(AT)*_*X*_-GT	12	12	12	ND	12	12	ND	12	12	12	12	12	12
MS7	AT-TA-*(TA)*_*X*_-GG	9	9	9	9	9	9	9	9	ND	9	9	ND	9
MS8	TGAC-*(AT)*_*X*_-GG	13	13	13	13	13	13	13	13	13	13	13	13	13
MS10	AGT-*(ACT)*_*X*_-*(AGA)*_*Y*_-*(TGA)*_*Z*_-CAA	6.14.9	6.14.9	6.14.9	ND	6.14.9	6.14.9	6.14.9	6.14.9	6.14.9	6.14.9	6.14.9	6.14.9	6.14.9
MS12	GC-*(GT)*_*X*_-GC	16	16	16	16	16	16	16	16	16	16	16	16	16
MS21	TG-(TACA)_3_-TACC-*(TACA)*_*X*_-TT	6	6	6	6	6	6	6	6	6	6	6	ND	6

^a^Microsatellite alleles are expressed as the number of repeats (X, Y and Z) in the motif sequences according to fragment size analysis and sequencing [20]. *ND*: Not determined.

### Histopathological features

Histological and immunohistochemical analyses were carried out in available tissues from 8 foetuses/lambs, of which 4 were positive to *N. caninum* PCR (samples 4, 12, 20 and 21) and 4 negative to both PCRs (samples 13, 14, 18 and 19).

**Figure 1:**
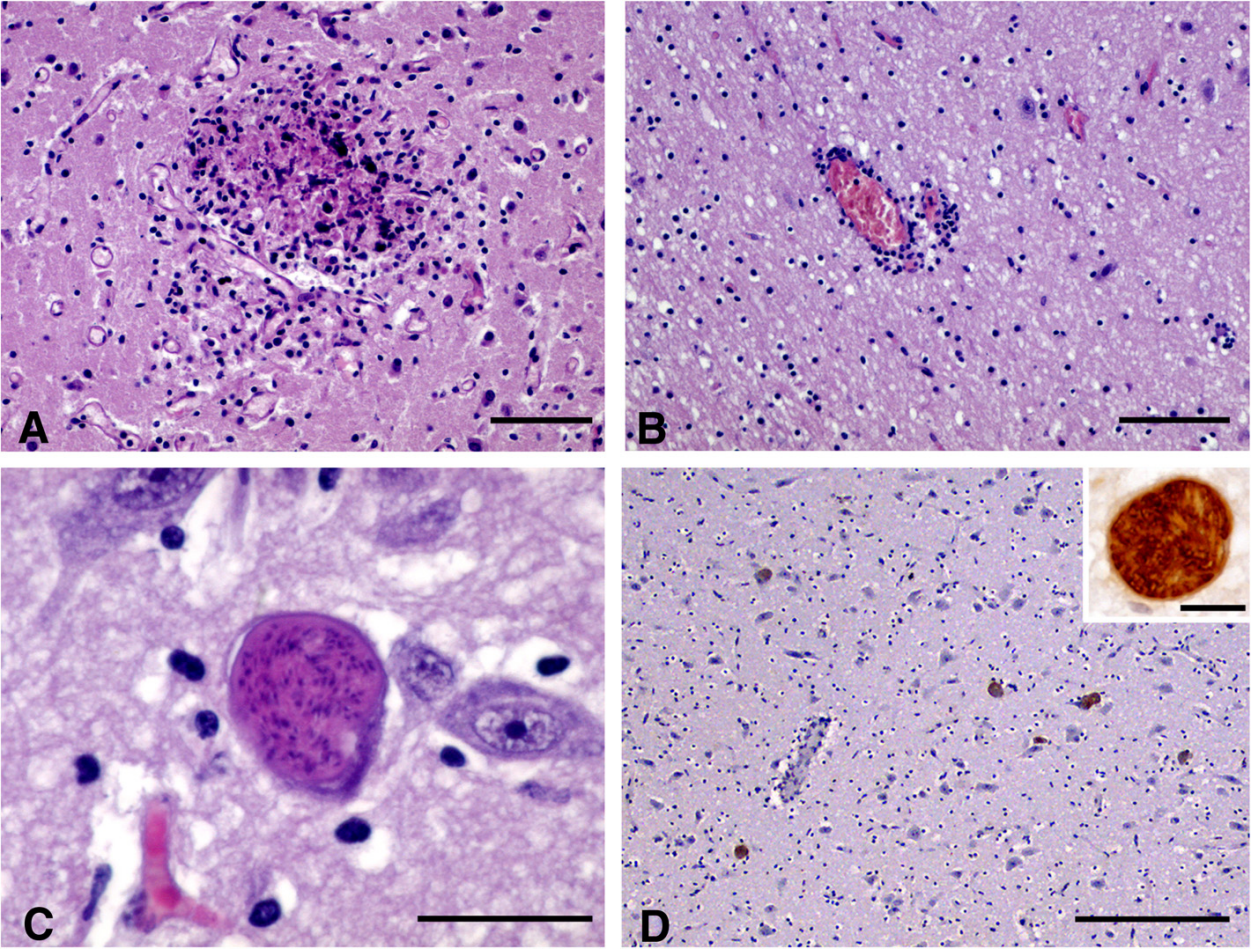
**Histological findings in foetus/lamb brains positive to** ***Neopora caninum*** **PCR. A)** Focus of gliosis with central area of coagulative to caseous necrosis in the grey matter (HE; Bar = 100 μm); **B)** Mild perivascular infiltration of mononuclear inflammatory cells in the white matter (HE; Bar = 200 μm); **C)** Intracellular cyst containing high number of bradyzoites. Note the absence of inflammation (HE; Bar = 25 μm); **D)** Immunohistochemical labelling of several protozoan tissue cysts in the grey matter. Note the absence of evident inflammatory infiltration in relation to the cysts (IHC; Bar = 300 μm) Inset: Detail of an immunolabelled tissue cyst (IHC; Bar = 15 μm).

Histological changes consistent with protozoal infections were observed in the brains of most foetuses/lambs testing positive by *N. caninum*-PCR (samples 4, 20 and 21) (Table 3). Specifically, the brain from foetus 4 showed multifocal necrotizing encephalitis with several randomly distributed foci of necrosis surrounded by microgliosis and occasional dystrophic mineralization (Figure 1A). A single small glial focus with central necrosis was detected in brain tissue from newborn lamb number 20, whereas samples from case 21 showed non purulent encephalitis characterized by scarce random glial foci, without necrosis, and mild perivascular infiltration of mononuclear cells (Figure 1B). Several tissue cysts with capsules and numerous internal bradyzoites were also seen throughout this brain (Figure 1C). Microscopic lesions were not detected in the brain from stillborn lamb number 12. Although brain cysts were only seen in one case (foetus 21) with HE staining, immunohistochemical labelling with anti-*N. caninum* tachyzoites serum enabled identification of parasite cysts that were randomly distributed within the grey matter of the brains from the 4 foetuses/lambs positive to *N. caninum* PCR analyzed (Table 3, Figure 1D).

Lesions consistent with infection by protozoa or parasite cysts were not evident in the livers, hearts or lungs from these PCR-positive foetuses/lambs. However, the only placenta examined (foetus number 4) showed several foci of coagulative necrosis, some of them with mineral deposition. Scant aggregates of mononuclear inflammatory cells were observed within the villous stroma of the placenta, usually close to the base of the cells. No parasitic form was found in the HE slides, but protozoan antigen was seen in association with the necrotic foci in slides labelled with anti-*N. caninum* antiserum.

In 3 out of the 4 PCR-negative lambs examined, non specific lesions were observed. Specifically, widespread congestion of white matter (samples 13 and 18) and purulent meningitis (sample 14) were observed.

## Discussion

*Neospora caninum* has been sporadically reported as a cause of reproductive disorders in sheep. However, it has traditionally been considered that this agent was less important than *T. gondii* in sheep [2,25]. In this study, in which the aetiology of the reproductive failure of a sheep flock was investigated, antibodies against *N. caninum* and/or *T. gondii* were detected, with a higher percentage of seropositivity in the case of *N. caninum*. Exposure to other infectious agents involved in ovine reproductive problems was disregarded, and we focused on determining the actual significance of the antibody levels against both protozoa detected in this flock. For this purpose, we assessed the relationship between seropositivity (to *N. caninum*, *T. gondii* or both protozoa) and reproductive performance. We also carried out molecular and histopathological analyses of foetuses and lambs for accurate determination of the involvement of both protozoa in the aetiology of the abortions and neonatal deaths in this flock.

In relation to the reproductive performance, we did not detect any differences between *T. gondii*-seropositive and seronegative sheep. However, it is well known that *T. gondii* induces foetopathy and abortion when sheep suffer primary infection during gestation [26], which is unlikely to have occurred in the flock under study, given the low levels of anti-*T. gondii* antibodies detected (close to the cut-off value) and the absence of seroconversion during the period of study. The serological results are consistent with the existence of antibodies remaining from a past infection, which, in turn, may explain the absence of reproductive disorders in the ewes that were seropositive to *T. gondii.* On the contrary, in the ewes that were seropositive to *N. caninum*, both the number of lambings and the rate of viable lambs per ewe were significantly lower than in seronegative sheep. Moreover, the anti-*N. caninum* antibody levels were very high (mean values were ten times higher than the cut-off value), reflecting the existence of an active infection that may cause reproductive failure. The reproductive output of sheep that were seropositive to both *T. gondii* and *N. caninum* was very similar to that displayed by sheep that were only seropositive to *N. caninum*, i.e. it was lower than that of the sheep that were either seronegative or seropositive to *T. gondii* only. This also supports the hypothesis that infection by *N. caninum* was the cause of the low reproductive performance of this flock. Nevertheless, very few animals were seropositive to both parasites, mainly in the second year of the study (*n* = 6), so that these results must be interpreted with caution.

The presence of antibodies in ewes does not provide conclusive diagnosis of foetal neosporosis and/or toxoplasmosis, which must be confirmed by analysis of foetuses/lambs by other techniques, such as PCR (to detect parasite DNA) and histology (to demonstrate protozoan associated lesions and/or parasite antigens). Nevertheless, analysis of foetuses/lambs is not usually performed in pasture-reared sheep flocks, because abortions often go undetected or are found autolyzed in the field. In our study, 21 foetuses/lambs were collected and analyzed by PCR. Histological and immunohistochemical analyses were able to be carried out in 8 of these. The PCR technique detected *N. caninum* DNA in the brains of 13 out of 14 descendants from sheep seropositive to this parasite. In addition, we observed parasite antigen and/or characteristic lesions, as previously described [2,7,27], in all *N. caninum*-DNA positive brains that were able to be analyzed. These results, which are concordant with those observed in bovine foetuses [28], demonstrate that the brain should be the organ of choice for the diagnosis of ovine neosporosis in aborted foetuses and neonatal lambs.

On the other hand, *N. caninum* DNA was detected in liver and heart from 3 of the 6 newborn lambs examined (numbers 5, 6 and 21), which contrasts with the findings for bovine foetuses, in which *N. caninum* is rarely detected in those organs in the third term of gestation [28]. This may be due to differences in the foetal immune response in the host species, although other factors related to the virulence of the parasite isolate cannot be ruled out and deserve further investigation.

With regard to *T. gondii*, no evidence of infection was found in the samples from the foetuses/lambs examined, which is consistent with the absence of specific antibodies in the dams. Moreover, the fact that seroconversion was not observed allows us to rule out the existence of infection of dams during gestation. Unfortunately, precolostral serum samples were not able to be collected for detection of antibodies in the offspring of seropositive dams.

Traditionally, ovine neosporosis has been associated with sporadic cases of abortion [25,29] with no serious impact on the productivity of the flocks [30]. Nevertheless, the findings of the present study conclusively demonstrate an association between infection by *N. caninum* and reproductive losses in sheep. However, the aetiology of reproductive failure is multifactorial and therefore the possible contribution of other factors (physical, chemical and biological) cannot be completely ruled out.

As in cattle, "endogenous" transplacental transmission, due to reactivation of latent infections during pregnancy, appears to be the main mechanism by which neosporosis was maintained in this sheep flock. This hypothesis was supported by the fact that there was a close association between seropositivity in dams and their daughters in the flock. Moreover, changes in serostatus were scarce, i.e. very few sheep became seropositive during the study, and their antibody levels rose just slightly above the cut-off. These results may be due to variations in antibody levels, as frequently observed in cattle, rather than to horizontal transmission in which higher antibody levels would be expected. Furthermore, no infections were detected in the guard dogs living alongside the sheep during the study period. On the other hand, we found only two closely related microsatellite multilocus genotypes (differing in only one repeat unit) in all clinical cases examined, which strongly suggests that *N. caninum* infection was maintained in the flock by clonal propagation, probably by 'endogenous' transplacental transmission rather than by ingestion of oocysts. Nevertheless, new research is required to characterize the *N. caninum* population circulating in sheep flocks and to determine the importance of horizontal transmission in this host species.

Finally, it should be noted that this study was conducted in a region where bovine neosporosis is highly prevalent, and therefore horizontal transmission of infection to the sheep flocks sharing pastures and water sources with cattle could occur. Nevertheless, in order to establish an epidemiological connection between both host species, the *N. caninum* genotypes that infect sheep and cattle must be identified and compared.
